# *In Vitro* and *In Vivo* Comparison of Selected Ga-68 and Zr-89 Labelled Siderophores

**DOI:** 10.1007/s11307-015-0897-6

**Published:** 2015-09-30

**Authors:** Milos Petrik, Chuangyan Zhai, Zbynek Novy, Lubor Urbanek, Hubertus Haas, Clemens Decristoforo

**Affiliations:** Institute of Molecular and Translational Medicine, Faculty of Medicine and Dentistry, Palacky University, Hnevotinska 5, CZ-77900 Olomouc, Czech Republic; Clinical Department of Nuclear Medicine, Medical University Innsbruck, Innsbruck, Austria; Laboratory of Growth Regulators, Centre of the Region Hana for Biotechnological and Agricultural Research, Institute of Experimental Botany AS CR & Palacky University, Olomouc, Czech Republic; Division of Molecular Biology/Biocenter, Medical University Innsbruck, Innsbruck, Austria

**Keywords:** Siderophores, Gallium-68, Zirconium-89, PET, Imaging

## Abstract

**Purpose:**

Some [^68^Ga]siderophores show promise in specific and sensitive imaging of infection. Here, we compare the *in vitro* and *in vivo* behaviour of selected Ga-68 and Zr-89 labelled siderophores.

**Procedures:**

Radiolabelling was performed in HEPES or sodium acetate buffer systems. Radiochemical purity of labelled siderophores was determined using chromatography. Partition coefficients, *in vitro* stability and protein binding affinities were determined. *Ex vivo* biodistribution and animal imaging was studied in mice.

**Results:**

Certain differences among studied siderophores were observed in labelling efficiency. Protein binding and stability tests showed highest stabilities and lowest protein binding affinities for Ga-68 and [^89^Zr]triacetylfusarinine C (TAFC). All studied Ga-68 and [^89^Zr]siderophores exhibited a similar biodistribution and pharmacokinetics in mice with the exception of [^89^Zr]ferrioxamine E (FOXE).

**Conclusions:**

Zr-89 and [^68^Ga]siderophores showed analogous *in vitro* and *in vivo* behaviour. Tested [^89^Zr]siderophores could be applied for longitudinal positron emission tomography (PET) studies of fungal infections and especially TAFC for the development of novel bioconjugates.

**Electronic supplementary material:**

The online version of this article (doi:10.1007/s11307-015-0897-6) contains supplementary material, which is available to authorized users.

## Introduction

Siderophores are high affinity, iron-selective chelators produced by almost all bacteria, fungi and some plants [1, 2]. They serve as carrier molecules transporting iron across the microbial cell membranes, thus representing an excellent source of iron for many microorganisms. Iron is an essential nutrient for all cells and plays an important role also in microbial virulence [3]. Under iron-restricted conditions, some microorganisms secrete siderophores into the environment where they collect iron from various sources and deliver it into the producing organism [4]. In some species, specialized siderophores are synthesized, which are not excreted and serve as a unique iron storage form. Siderophores and their analogues have found wide application in agriculture and in medicine [5]. In medicine, siderophores have shown potential in applications like selective drug delivery; treatment of diseases, e.g., thalassemia, malaria and sickle cell disease; cancer therapy and imaging cancer and infection [5, 6].

Some Gallium-68 (Ga-68) labelled siderophores have demonstrated their use as specific agents for imaging *Aspergillus* infection [7]. Gallium (Ga^3+^; ionic radius = 62 pm) and iron (Fe^3+^; ionic radius = 64 pm) are structurally similar and have similar coordination properties. Ga-siderophore complexes are recognized by cellular transporters and receptors on bacterial cells allowing the complex to enter inside the microbial cell. Ga-68 is a radioactive isotope of gallium and has in recent years gained a lot of attention in the field of nuclear medicine for molecular imaging using positron emission tomography (PET) [8, 9]. It is a short-lived positron emitter (half-life = 67.7 min) that can be produced from a long shelf-life and cost-effective [^68^Ge]/[^68^Ga] generator system. The relatively short half-life of Ga-68 can be a limitation especially in longitudinal studies. This deficit can be resolved using longer-lived positron-emitting radionuclides such as Cu-64, I-124, Y-86, Nb-90 or Zr-89 [10].

The interest in Zirconium-89 (Zr-89) has increased over the last years as it displays almost ideal properties allowing imaging of biological processes at late time-points after the tracer application [11]. Even though Zr-89 has comparably low positron abundance and due to the long half-life results in higher radiation dose, it allows long-term follow-up especially of slowly accumulating biomolecules such as antibodies, nanoparticles and other large biomolecules both for preclinical and clinical applications, thereby complementing Ga-68 with its limitations of a very short half-life. Zr-89 can be produced in a cyclotron in large quantities and at high radionuclidic purity (99.99 %) [12]. It decays with a half-life of 78.4 h via both positron emission (23 %) and electron capture (77 %). The relatively low positron energy of 396.9 keV results in high image resolution [13]. The preferred oxidation state of zirconium is +4 with an ionic radius of 72 pm, also similar to the ferric iron.

**Fig. 1:**
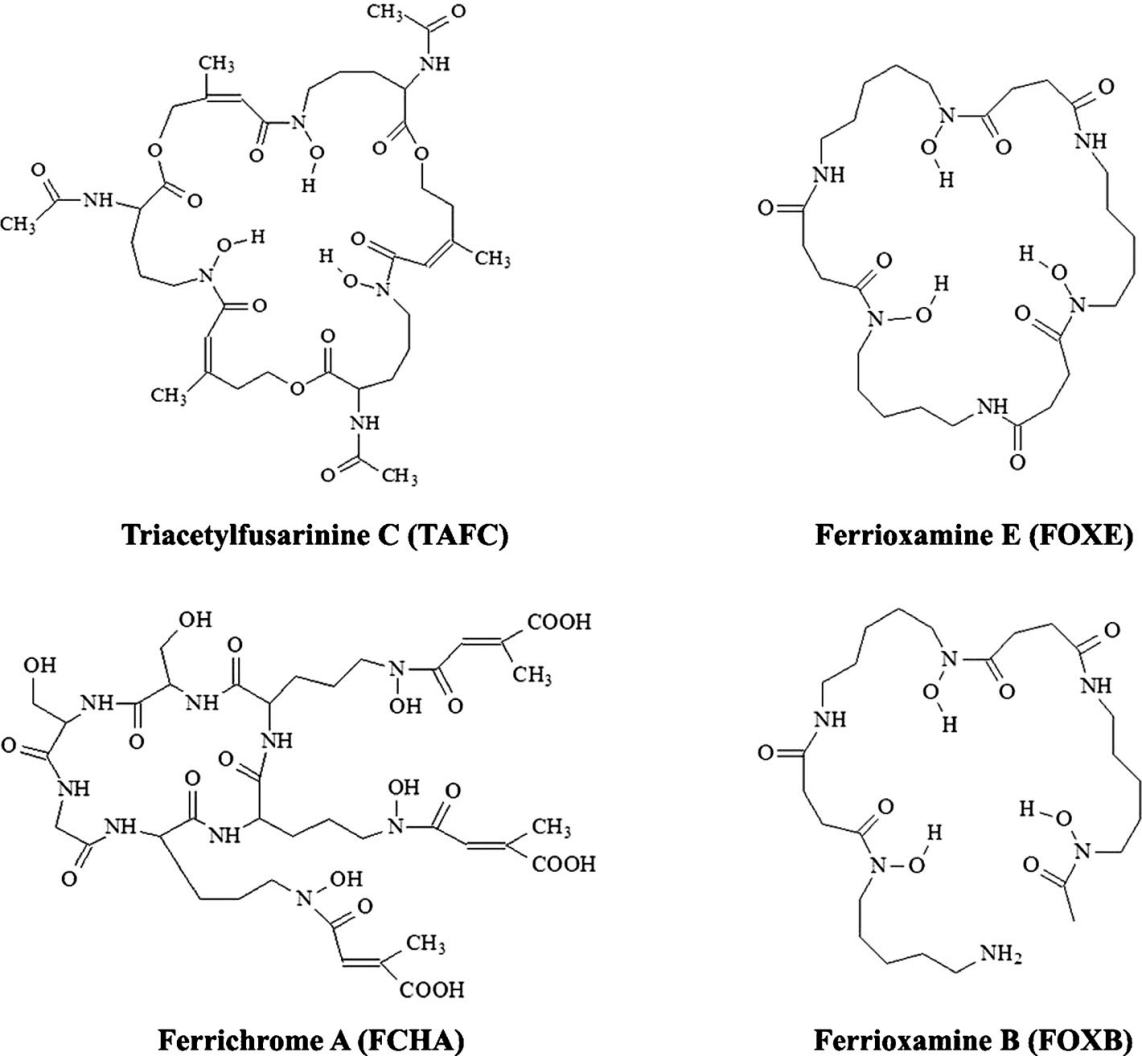
Structures of studied siderophores.

In our previous studies, we have shown that different siderophores can be labelled with Ga-68 [14]. We have also demonstrated that [^68^Ga]triacetylfusarinine C (TAFC) and [^68^Ga]ferrioxamine E (FOXE) are able to detect *Aspergillus fumigatus* infection in a rat infection model using PET imaging [7, 15] and that [^68^Ga]TAFC is highly specific to *A. fumigatus in vitro* [16]. Desferrioxamine B (FOXB), a hydroxamate siderophore, is the most commonly used bifunctional chelator for coordination of Zr-89^4+^. Several preclinical proof-of-principle studies have been conducted using FOXB to label antibodies with Zr-89, but the *in vivo* stability of this complex remains an issue [17]. Our aim in this study was to evaluate the potential of alternative siderophores for radiolabelling with Zr-89 and as imaging agents for *Aspergillus* infections. We also wanted to investigate any potential differences between the resulting Zr-89 complexes and the respective Ga-68 complexes; the differences in charge (Zr^4+^ vs. Ga^3+^) could impact complex stability and other properties such as lipophilicity. We have investigated this issue and report here the *in vitro* and *in vivo* properties of selected Zr-89 and Ga-68 labelled siderophores (Fig. 1).

## Materials and Methods

### Chemicals

All commercially available reagents were used as supplied with no further purification. Siderophores were purchased from EMC microcollections GmbH (Tubingen, Germany). [^68^Ga]Cl_3_ was eluted from a [^68^Ge]/[^68^Ga] generator (Eckert & Ziegler Eurotope GmbH, Berlin, Germany) with 0.1 N HCl using the fractionated elution approach. Zr-89 in 1 M oxalic acid was produced by the BV Cyclotron VU (Amsterdam, The Netherlands) and distributed by PerkinElmer (Boston, USA).

### Siderophores Radiolabelling and Quality Control

Ga-68 labelled siderophores were prepared as previously described [14]. Radiolabelling of siderophores with Zr-89 was performed using 9–11 μl of Zr-89 in 1 M oxalic acid (7–12 MBq) and neutralized with 10 μl 1 M Na_2_CO_3_, then mixed with 100 μl of HEPES buffer (0.5 M, pH = 7) and 20–50 μl of an aqueous solution of desferrisiderophores (1 μg/μl). The reaction mixture was allowed to react at room temperature (RT) for 90 min. Alternatively, direct incubation of 9–11 μl Zr-89 in 1 M oxalic acid with 30–40 μl of sodium acetate (155 mg/ml in water) mixed with 20–50 μl of desferrisiderophores dissolved in water (1 μg/μl) was performed. Incubation times (15–20 min) and temperature (RT or 80 °C) were varied. Radiochemical purity (RCP) of labelled siderophores was determined on reverse-phase high-performance liquid chromatography (RP-HPLC) using a gradient system as described previously [15]. [^89^Zr]siderophores were additionally analysed by thin-layer chromatography (ITLC-SG/50 mM EDTA), in which [^89^Zr]siderophores remained at the origin and unbound Zr-89 moved with the solvent front.

### *In Vitro* Characterization of Radiolabelled Siderophores

#### Partition Coefficient Determination

Radiolabelled siderophore in 0.5 ml phosphate-buffered saline (PBS) pH = 7.4 was added to 0.5 ml octanol and the mixture was vigorously vortexed for 15 min. The aqueous and organic solvents were separated by centrifugation and 50 μl aliquots of both layers were collected and measured in the automatic gamma counter (WIZARD^2^; PerkinElmer, Waltham, USA). Log *P* values were calculated from obtained data (mean of *n* = 6).

#### Protein Binding

Protein binding studies were performed by incubating radiolabelled siderophore (1–2 μg/150 μl) in fresh human serum (850 μl) and in PBS (850 μl) (control) at 37 °C for various time points (30, 60 and 120 min for [^68^Ga]siderophores and 1, 4 and 24 h for [^89^Zr]siderophores). After incubation, 25 μl of the sample was separated by size-exclusion chromatography (MicroSpin™ G-50 Columns; Sephadex G-50 (GE Healthcare, Buckinghamshire, UK)) by centrifugation at 2000×*g* for 2 min. Protein binding of radiolabelled siderophores was determined by measuring the activity distributed between the column (non protein-bound) and the eluate (protein-bound) using the automatic gamma counter.

#### Stability Studies

The *in vitro* stability of radiolabelled siderophores (3–6 μg/100 μl) was tested in fresh human serum (300 μl) and a 6 mM solution of diethylenetriaminepentaacetic acid (DTPA) pH = 7 (100 μl) at 37 °C at several time points (30, 60 and 120 min for [^68^Ga]siderophores and 1, 4 and 24 h for [^89^Zr]siderophores). After incubation, human serum samples were precipitated with acetonitrile or ethanol, centrifuged (2200×*g*, 3 min) and the supernatant analyzed. Degradation of the radiolabelled siderophore complexes was evaluated by RP-HPLC. Samples of the DTPA solutions were directly injected onto the HPLC.

### Animal Experiments

All animal experiments were conducted in accordance with regulations and guidelines of the Czech Animal Protection Act (No. 246/1992), and with the approval of the Czech Ministry of Education Youth and Sports (MSMT-18933/2013-1), and the institutional Animal Welfare Committee of the Faculty of Medicine and Dentistry of Palacky University in Olomouc. The studies were performed using female Balb/c mice (Anlab, Prague, Czech Republic).

### Biodistribution in Balb/c Mice

Biodistibution of radiolabelled siderophores was studied in female Balb/c mice (8 weeks old). Ga-68 and Zr-89 labelled siderophores (1–2 MBq/mouse, corresponding to 0.1–0.2 μg of siderophore per mouse) were injected retro-orbitally (r.o.). Animals were sacrificed by cervical dislocation 30 and 90 min post-injection (p.i.). Organs and tissues (blood, spleen, pancreas, stomach, intestine, kidneys, liver, heart, lung, muscle and femur) were removed and weighed. The amount of radioactivity in the samples was measured in an automatic gamma counter. Results were expressed as percentage of injected dose per gram organ (%ID/g).

### PET Imaging of Radiolabelled Siderophores in Balb/c Mice

PET and computed tomography (CT) images were acquired with an Albira PET/SPECT/CT small animal imaging system (Bruker Biospin Corporation, Woodbridge, CT, USA). Female Balb/c mice were r.o. injected with radiolabelled siderophores in a dose of 5–10 MBq corresponding to 0.5–1 μg of siderophore per mouse. Animals were anaesthetized with isoflurane (FORANE, Abbott Laboratories, Abbott Park, IL, USA) (2 % flow rate) and positioned prone head first in the Albira system before the start of imaging. Static PET/CT imaging was carried out 5, 30 and 90 min p.i. for both Ga-68 and Zr-89 labelled siderophores. Animals injected with [^89^Zr]siderophores were also imaged 4 h p.i. A 5-min PET scan (axial FOV 148 mm) was performed, followed by a CT scan (axial FOV 65 mm, 45 kVp, 400 μA, at 600 projections). Dynamic imaging was carried out immediately after the injection of radiolabelled siderophore for 90 min (5 min PET scan per frame). Scans were reconstructed with the Albira software (Bruker Biospin Corporation, Woodbridge, CT, USA) using the maximum likelihood expectation maximization (MLEM) and filtered backprojection (FBP) algorithms. After reconstruction, acquired data was viewed and analyzed with PMOD software (PMOD Technologies Ltd., Zurich, Switzerland). 3D images were obtained using VolView software (Kitware, Clifton Park, NY, USA). Animal imaging was performed for all studied siderophores except for ferrichrome A (FCHA).

## Results

### Radiochemistry

The labelling conditions as well as labelling efficiency were slightly different for studied desferrisiderophores. Desferritriacetylfusarinine C (TAFC) and desferriferichrome A (FCHA) were labelled with Ga-68 in sodium acetate buffer at room temperature for 15 min with an RCP greater than 98 %. Radiolabelling of desferrioxamine E (FOXE) and desferrioxamine B (FOXB) with Ga-68 was performed in sodium acetate buffer at 80 °C for 20 min with an RCP greater than 98 %. Zr-89 labelling of all studied siderophores using Na_2_CO_3_ for neutralization followed by incubation with the siderophore in HEPES buffer (pH = 7) required incubation times of more than 90 min at RT with the RCP greater than 98 %. Interestingly, direct incubation of [^89^Zr]oxalate with the corresponding siderophore in sodium acetate buffer at 80 °C for 20 min resulted in equivalent results with a radiochemical purity of not less than 90 % as determined by HPLC (Suppl Fig. 1) and ITLC-SG. This was considered as sufficient for this initial evaluation of [^89^Zr]siderophores, and the methods using sodium acetate buffer for Ga-68 as well as for Zr-89 labelling of siderophores were used for further *in vitro* and *in vivo* experiments.

### *In Vitro* Characterization

All studied siderophores showed hydrophilic properties with partition coefficients (log *P*) ranging from −1.65 to −3.56. FOXE and FCHA displayed very similar log *P* values for both Ga-68 and Zr-89 complexes, while TAFC and FOXB displayed more lipophilic character when labelled with Zr-89. Protein binding values did not exceed 11 % for [^68^Ga]siderophores (incubation time 120 min), whereas [^89^Zr]FOXE and [^89^Zr]FCHA showed 16.6 and 31.1 % of protein binding after 24-h incubation period. All the [^68^Ga]siderophores were stable *in vitro*, with the exception of [^68^Ga]FOXB (Table 1). The stability studies of [^89^Zr]siderophores (Table 2) displayed some differences compared to [^68^Ga]siderophores. In particular, [^89^Zr]FCHA was less stable as compared to its Ga-68 labelled counterpart whereas [^89^Zr]FOXB revealed higher stability in all examined media.

**Table 1:** *In vitro* characterization of studied Ga-68 labelled siderophores. Log *P*, protein binding (expressed as % of protein bound activity of the total activity used) and stability (in human serum and 6 mM DTPA) of [^68^Ga]TAFC, [^68^Ga]FOXE, [^68^Ga]FCHA and [^68^Ga]FOXB

[^68^Ga]siderophore	Log *P* (mean ± SD, *n* = 6)	Incubation time (min)	Protein binding (%) (mean, *n* = 2)	Stability in human serum (%) (*n* = 1)	Stability in DTPA solution (%) (*n* = 1)
[^68^Ga]TAFC	−2.59 ± 0.15	30	0.47	99.9	85.0
		60	0.76	99.9	84.7
		120	1.21	99.9	81.8
[^68^Ga]FOXE	−1.65 ± 0.03	30	0.27	99.9	94.3
		60	0.24	99.9	93.8
		120	0.53	99.9	93.2
[^68^Ga]FCHA	−3.24 ± 0.07	30	5.12	97.4	97.7
		60	6.98	97.8	95.7
		120	4.21	98.5	92.3
[^68^Ga]FOXB	−3.56 ± 0.17	30	7.67	74.1	60.1
		60	10.29	72.0	54.5
		120	10.83	75.4	52.9

**Table 2:** *In vitro* characterization of studied Zr-89 labelled siderophores. Log *P*, protein binding (expressed as % of protein bound activity of the total activity used) and stability (in human serum and 6 mM DTPA) of [^89^Zr]TAFC, [^89^Zr]FOXE, [^89^Zr]FCHA and [^89^Zr]FOXB

[^89^Zr]siderophore	Log *P* (mean ± SD, *n* = 6)	Incubation time (min)	Protein binding (%) (mean, *n* = 2)	Stability in human serum (%) (*n* = 1)	Stability in DTPA solution (%) (*n* = 1)
[^89^Zr]TAFC	−1.95 ± 0.03	1	0.99	98.08	99.51
		4	0.34	98.72	99.50
		24	1.37	98.50	99.50
[^89^Zr]FOXE	−1.91 ± 0.04	1	3.27	98.07	99.70
		4	5.86	99.70	99.50
		24	16.59	98.34	99.70
[^89^Zr]FCHA	−3.28 ± 0.37	1	2.70	92.76	68.70
		4	6.24	85.42	65.27
		24	31.05	77.38	54.22
[^89^Zr]FOXB	−3.01 ± 0.07	1	1.63	99.61	91.56
		4	2.05	99.70	90.39
		24	1.84	99.53	87.10

### *Ex Vivo* Biodistribution in Balb/c Mice

**Fig. 2:**
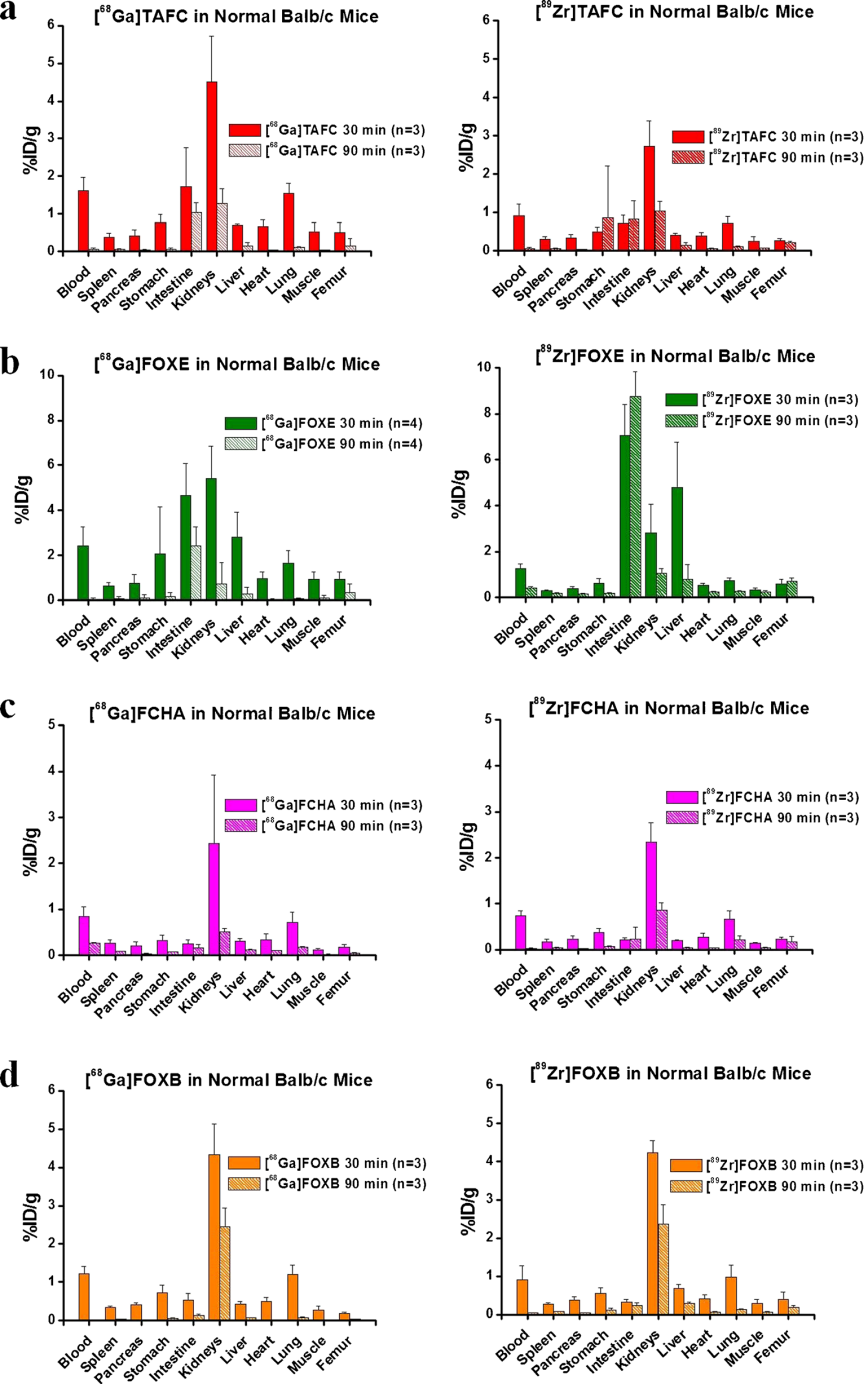
*Ex vivo* biodistribution of studied radiolabelled siderophores in Balb/c mice 30 and 90 min p.i. Data are presented as percentage of injected dose per gram organ (%ID/g ± SD; *n* = 3).

Ga-68 and Zr-89 labelled TAFC, FCHA and FOXB showed virtually identical *ex vivo* biodistribution in Balb/c mice. All six compounds were rapidly excreted via the renal system and showed minimal retention in blood and the various organs. [^68^Ga]FOXE revealed similar biodistribution with slightly higher radioactivity values in gastrointestinal tract compared to the other radiolabelled siderophores, whereas [^89^Zr]FOXE showed significantly higher radioactivity accumulation in the intestines and liver. In all cases, [^89^Zr]siderophores displayed slightly higher bone uptake compared to their Ga-68 labelled counterparts, but always lower than 1 %ID/g, whereas there was a trend of higher blood activity for Ga-68 vs. [^89^Zr]compounds, both indicating the behaviour of the free radionuclides. *Ex vivo* biodistribution data are presented in Fig. 2.

### Small Animal PET Imaging

**Fig. 3:**
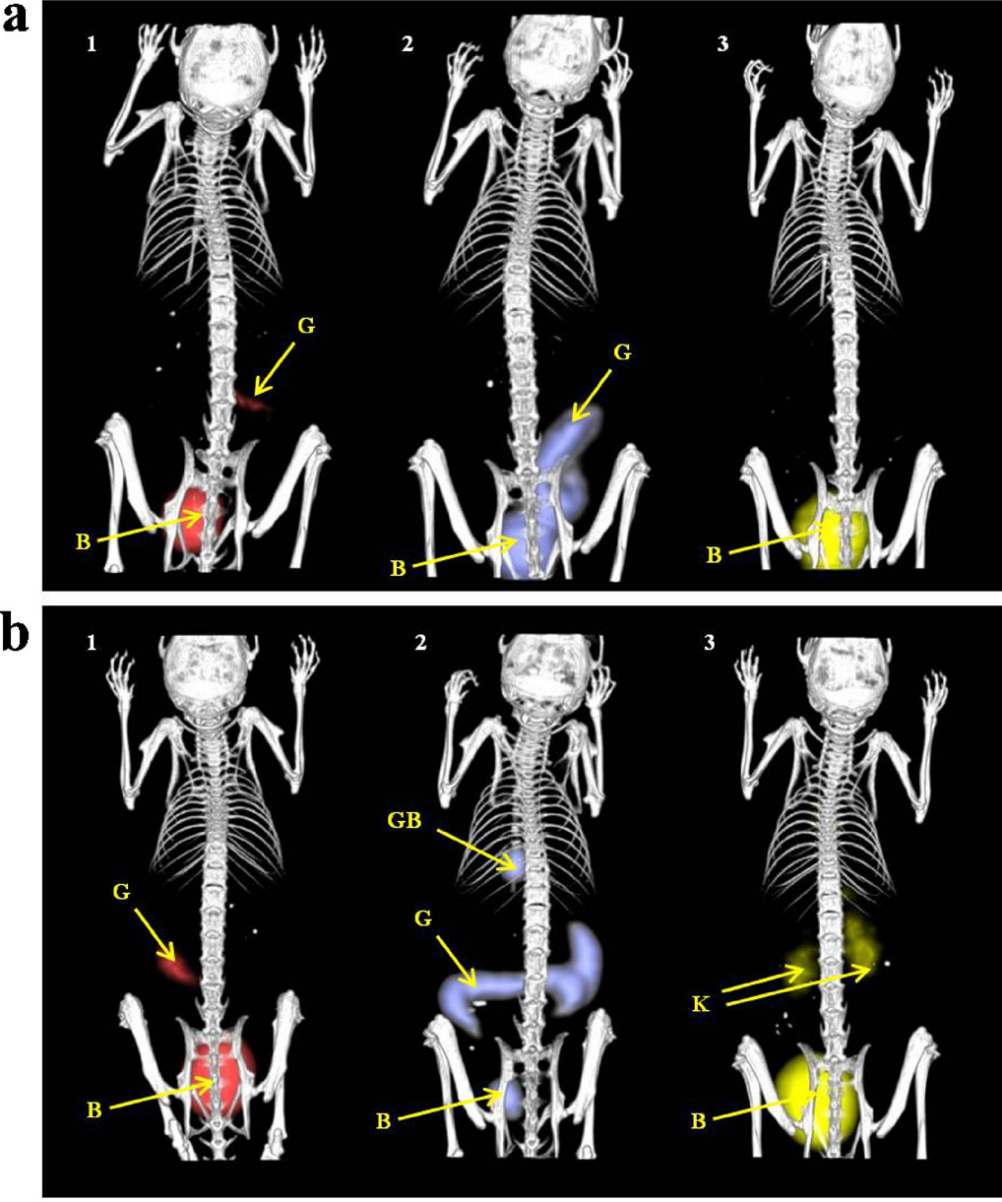
3D volume rendered images of static μPET/CT imaging of [^68^Ga]siderophores (**a—**TAFC = 1, FOXE = 2, FOXB = 3) and [^89^Zr]siderophores (**b**—TAFC = 1, FOXE = 2, FOXB = 3) 90 min p.i. (supine position; injected dose, 5–10 MBq; anaesthesia, 2 % isoflurane; scan duration, 5 min PET scan followed by 20 min CT scan; *B*—bladder, *G*—gastrointestinal tract, *GB*—gall bladder, *K*—kidney).

**Fig. 4:**
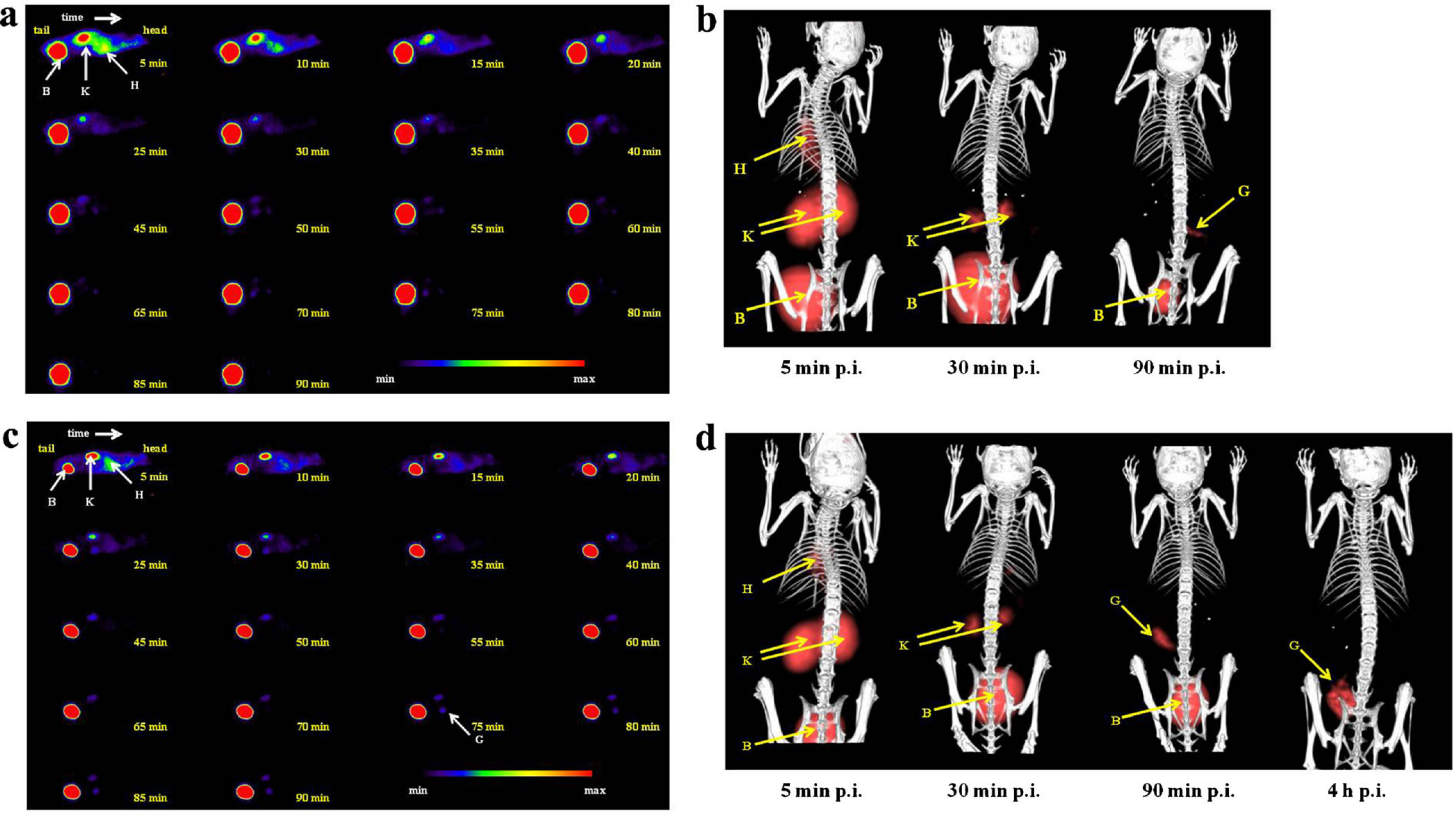
μPET/CT imaging of [^68^Ga]TAFC and [^89^Zr]TAFC in Balb/c mice: dynamic PET imaging of [^68^Ga]TAFC (**a**), static PET/CT imaging of [^68^Ga]TAFC (**b**), dynamic PET imaging of [^89^Zr]TAFC (**c**), static PET/CT imaging of [^89^Zr]TAFC (**d**). (Sagittal slices (**a**, **c**) and 3D volume rendered images (**b**, **d**); injected dose, 5–10 MBq; anaesthesia, 2 % isoflurane; scan duration, dynamic imaging = 5 min PET scan per frame (18 frames); static imaging = 5 min PET scan followed by 20 min CT scan; *B*—bladder, *G*—gastrointestinal tract, *H*—heart, *K*—kidney).

MicroPET imaging of Balb/c mice injected with Ga-68 and Zr-89 labelled TAFC and FOXB showed rapid clearance from the bloodstream with the major excretion route via kidneys. Both pairs of radiolabelled siderophores displayed very similar kinetics and biodistribution (Figs. 3 and 4). Significantly different *in vivo* behaviour was observed for the [^68^Ga]/[^89^Zr]FOXE pair. [^68^Ga]FOXE was quickly eliminated mainly via the kidneys with certain part of radioactivity excreted via the gastrointestinal tract, while [^89^Zr]FOXE revealed much slower kinetics and significant hepatobiliary elimination. Animal imaging data confirmed and supplemented the data from *ex vivo* biodistribution studies. The representative imaging data is presented in Figs. 3 and 4.

## Discussion

Until recently, the majority of preclinical and clinical research has focused on the development of PET radiopharmaceuticals using conventional PET radionuclides such as C-11, N-13, O-15 and F-18 [18]. This has changed with the increased availability and production of non-conventional positron-emitting radionuclides such as Cu-64, Ga-68, Y-86, Zr-89 and I-124. Recently, Ga-68 and Zr-89 have generated significant interest in the nuclear medicine community with a concomitant increase in the use of Ga-68 labelled peptides for PET imaging of neuroendocrine tumours and Zr-89 imunoPET [8, 19].

The radiolabelling of biomolecules with Zr-89 is usually performed by the desferrioxamine B (FOXB) chelator, commercially available as Desferal. FOXB is a hexadentate siderophore containing three hydroxamate groups for chelating metals and a primary amine functional group for conjugation to a biomolecule. It is a chelating agent for several metal ions besides Zr-89 [20]. Zr-89 radiolabelling of biomolecules using the FOXB chelator has shown that the *in vivo* stability of this complex remains an issue. This instability has been observed in several preclinical studies with uptake of Zr-89 in bones [21, 22]. While many investigators have focused on the modification of the linkage between the FOXB and the biomolecule [23, 24], others have attempted to improve the chelator itself [25–29]. Despite these attempts, a novel high-stability Zr^4+^ ligand which would minimize the uptake of liberated Zr^4+^ in the bone and other non-targeted tissue is warranted.

In this study, we have investigated the *in vitro* and *in vivo* behaviour of selected hydroxamate siderophores labelled with Zr-89 to evaluate their potential to be used as novel Zr-89 chelators and to compare their *in vitro* and *in vivo* properties with their Ga-68 labelled counterparts. We note that studied siderophores are only hexadentate ligands and cannot coordinatively saturate Zr^4+^ that can bind up to eight ligand atoms. Therefore, it is to be expected that the additional positive charge of Zr^4+^ will have a pronounced effect on chemical and biological properties of small chelators such as siderophores. However, the complexation of Zr^4+^ is affected not only by number of coordinating atoms but also by linear vs. cyclic character, optimal ring size and appropriate geometry of the ligand [26].

Triacetylfusarinine C (TAFC) and ferrioxamine E (FOXE) are macrocyclic hydroxamate siderophores with the ring size of 35 (TAFC) and 33 (FOXE) atoms, which is deemed to be in the appropriate range for Zr^4+^ chelation to preserve the optimal spatial orientation [25]. These siderophores were chosen due to their successful application as Ga-68 labelled tracers in the imaging of *Aspergillus* infection in rats [7]. We aimed to further test the ability of Zr-89 to label these promising infection imaging agents in order to enlarge their imaging time frame for imaging *Aspergillus* infection, e.g., for treatment monitoring. Ferrichrome A (FCHA) and FOXB are representatives of linear hydroxamate siderophores. FOXB was selected as a standard chelating agent used in nuclear medicine for Zr-89 labelling.

All siderophores that were tested could be labelled to near-completeness with both Ga-68 and Zr-89 using established labelling protocols. Both Ga-68 and Zr-89 labelled siderophores showed hydrophilic properties with [^89^Zr]siderophores having a tendency to be slightly more lipophilic. This is rather surprising, as the binding of Ga^3+^ results in a neutral molecule, whereas Zr^4+^ introduces an additional positive charge. Significant differences among studied siderophores were observed mainly in protein binding and *in vitro* stability. [^68^Ga]TAFC, [^68^Ga]FOXE and [^68^Ga]FCHA displayed identical or lesser protein binding abilities than their Zr-89 labelled counterparts, while [^68^Ga]FOXB revealed higher protein binding values in comparison with [^89^Zr]FOXB. It could be speculated that the change of cation in the complex with difference in charge leads to a more amphiphilic character resulting in higher binding to plasma proteins or possibly phospholipids, but more experimental data would be required to support such a hypothesis. Ga-68 labelled cyclic hydroxamate siderophores (TAFC and FOXE) and [^68^Ga]FCHA were very stable in all tested media, whereas [^68^Ga]FOXB showed pronounced *in vitro* instability. The *in vitro* stability of studied [^89^Zr]siderophores was different in some aspects. For instance, [^89^Zr]FOXB displayed a much higher *in vitro* stability than [^68^Ga]FOXB. *Ex vivo* biodistribution and small animal imaging of all Ga-68 and Zr-89 labelled siderophores injected into Balb/c mice displayed similar pharmacokinetics and minimal accumulation of radioactivity in blood and other organs and tissues with the exception of [^89^Zr]FOXE, which caused significant retention of radioactivity in the gastrointestinal tract. In all cases, [^89^Zr]siderophores displayed slightly higher (90 min p.i.), but still very low, retention of radioactivity in bones, indicating favourable *in vivo* stability in the studied time intervals. The differences in the behaviour of Ga-68 and the corresponding [^89^Zr]siderophores could be explained by the different charge of the two radio-metal ions, resulting in a positively charged complex for hydroxamate coordinations for Zr in contrast to a neutral overall charge in the case of Ga. Even though there was no obvious instability of Zr-89 compounds, further investigations are required especially in the view of a higher stability of Zr-complexes by using octacoordinating ligands [25, 28].

The overall comparable properties of Ga-68 and Zr-89 labelled siderophores (despite the differences of Ga and Zr in terms of charge, coordination chemistry and ionic radius) warrant further investigation of the targeting behaviour of Zr-89 labelled siderophores. This is of high interest in the application of the radiolabelled siderophores in the imaging of infection, in particular, infection by invasive fungal pathogens. The preclinical monitoring of therapy could benefit from a radionuclide that has a longer half-life, as it would enable longer imaging times (from minutes to days). However, there is no data available so far that proves that [^89^Zr]siderophores such as TAFC are recognized by the specific transporters of microorganisms in the same way as their Ga-68 labelled counterparts [7, 15]. Further studies in this respect are ongoing. The other application could lie in the development of novel, more stable [^89^Zr]bioconjugates, coupling siderophores to, e.g., receptor of antigen targeting sequences. Here, the difference of Zr-89 vs. Ga-68 labelling will not result in a difference in targeting properties, but may result in different pharmacokinetics due to changes in charge and resulting lipophilicity. In this regard, preliminary studies of fusarinine C (FSC)-based bioconjugates that have been described recently for Ga-68 labelling [30] and Zr-89 labelling [31] showed excellent *in vivo* stabilities and tumour targeting.

## Conclusion

We have shown in this study that a variety of siderophores can be labelled with Ga-68 and Zr-89. Ga-68 and [^89^Zr]siderophores displayed analogous, but not fully identical *in vitro* and *in vivo* behaviour. Ga-68^3+^ seems to be the more suitable radionuclide for labelling of hexadentate hydroxamate siderophores. Nevertheless, [^89^Zr]TAFC showed favourable properties for potential longitudinal *Aspergillus* infection imaging using the animal model from our previous studies, and also availability as a model structure for preparation of novel bioconjugates.

## Electronic Supplementary Material

Below is the link to the electronic supplementary material.

ESM 1 (PDF 408 kb)
