# Supplementary material for: In Vitro and In Vivo Comparison of Selected Ga-68 and Zr-89 Labelled Siderophores
Source: Mol Imaging Biol. 2015 Sep 30;18:344–52. doi: 10.1007/s11307-015-0897-6 (PMC4870302; doi:10.1007/s11307-015-0897-6)
Supplement: Supplementary file 1 — (PDF 408 kb) [file 11307_2015_897_MOESM1_ESM.pdf]

Supplemental Material

In Vitro and *In Vivo* Comparison of Selected Ga-68 and Zr-89 Labelled Siderophores

Journal: Molecular Imaging and Biology

Milos Petrik[1], Chuangyan Zhai[2], Zbynek Novy[1], Lubor Urbanek[3], Hubertus Haas[4] and Clemens Decristoforo[2]

[1]Institute of Molecular and Translational Medicine, Faculty of Medicine and Dentistry, Palacky University, Olomouc, Czech Republic

[2]Clinical Department of Nuclear Medicine, Medical University Innsbruck, Innsbruck, Austria

[3]Laboratory of Growth Regulators, Centre of the Region Hana for Biotechnological and Agricultural Research, Institute of Experimental Botany AS CR & Palacky University, Olomouc, Czech Republic

[4]Division of Molecular Biology/Biocenter, Medical University Innsbruck, Innsbruck, Austria

Corresponding author:

Milos Petrik

Institute of Molecular and Translational Medicine, Hnevotinska 5, CZ-77900 Olomouc, Czech Republic

Email: milospetrik@seznam.cz

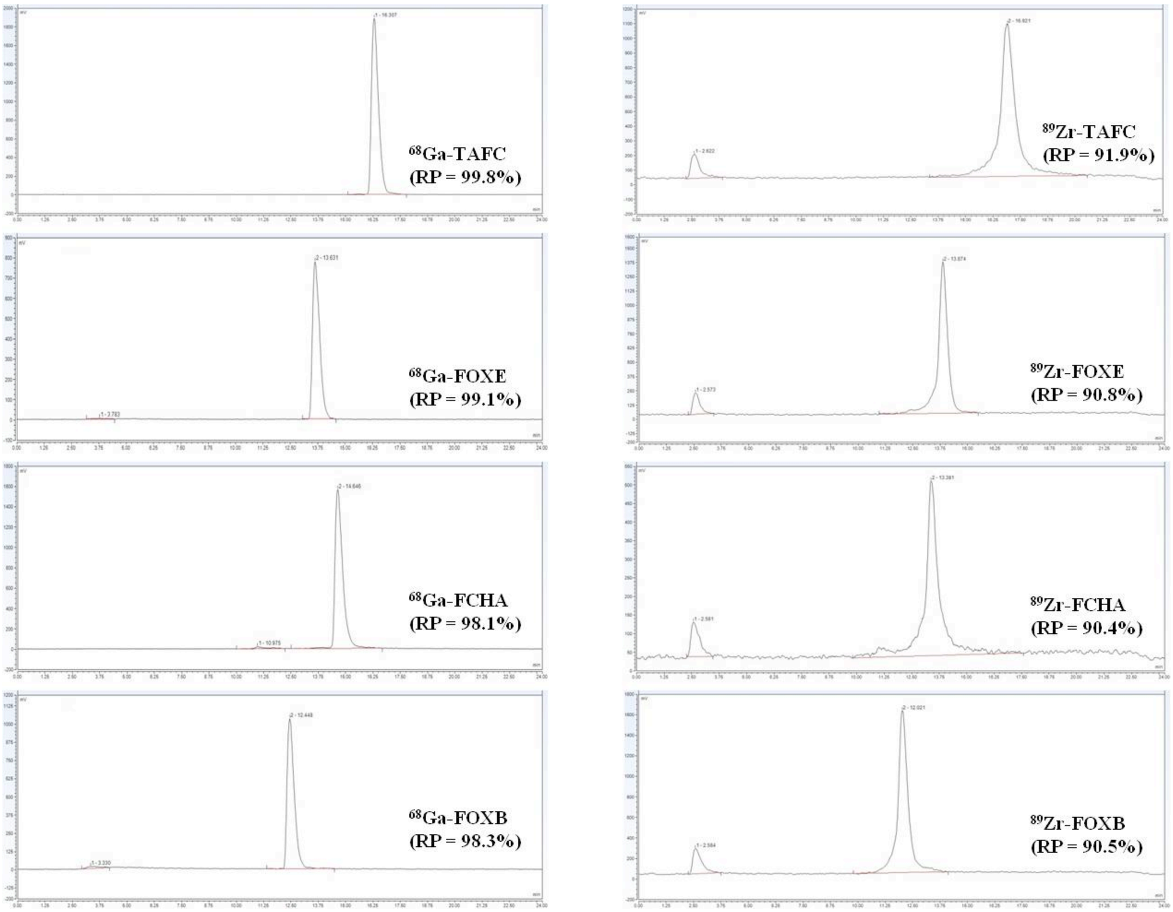

Supplemental Fig. 1 Examples of high-performance liquid chromatography-radiochromatograms of studied siderophores with respective radiochemical purity (RP) in %.
